# Practice analysis of junior doctors in Ethiopia: implications for strengthening medical education, practice and regulation

**DOI:** 10.1186/s41256-018-0086-7

**Published:** 2018-11-08

**Authors:** Daniel Dejene, Tegbar Yigzaw, Samuel Mengistu, Zerihun Wolde, Abiy Hiruy, Damtew Woldemariam, Miftah Awol

**Affiliations:** 1 Jhpiego, Kirkos Subcity, Box 2881, Code, 1250 Addis Ababa, PO Ethiopia; 2 University of Maryland Prince George’s Hospital center, Cheverly, Maryland USA; 3 Federal Ministry of Health, Addis Ababa, Ethiopia; 4 Ethiopian Medical Association, Addis Ababa, Ethiopia

**Keywords:** Practice analysis, Junior medical doctors, Ethiopia

## Abstract

**Background:**

A high performing physician workforce is critical to attain nationally set health sector goals. Ethiopia has expanded training of medical doctors. However, little is known about junior doctors’ performance. Understanding medical practice is essential to inform medical education and practice, establish licensure examination and guide workforce management decisions. We conducted a practice analysis study to identify gaps in Ethiopian medical education and practice, and to determine composition of subjects in national licensing examination.

**Methods:**

We conducted a cross-sectional study with national representative sample of junior doctors. After calculating a sample size of 198, we used a two-stage stratified cluster sampling method to select study participants. We collected data using a structured questionnaire comprising 222 tasks. Study participants reported in interviews on frequency of, competence at, and importance of doing each task for improved health outcome. We developed proportions, averages, graphs and tables. Using the results of practice analysis and experts’ ratings, relative weights of subjects in the national licensing examination for medical undergraduates were determined.

**Results:**

A total of 191 junior doctors participated. Most were males (74.6%) and had less than 2 years of experience (69.8%). Junior doctors frequently performed tasks of internal medicine and pediatrics. Their participation in obstetrics and gynecology, ophthalmology, psychiatry and dentistry services was infrequent. Junior doctors had competency gaps to conduct clinical procedures, research and health programming tasks. Practice analysis results and expert ratings generated comparable recommendations for composition of a national licensing examination, with more than three-quarters of the items focusing on internal medicine, pediatrics, surgery, obstetrics and gynecology, and public health.

**Conclusion:**

Junior doctors in Ethiopia rarely managed psychiatry, ophthalmology and dental patients. They had competence gaps in clinical procedures, research and health programming skills. The findings have implications for establishing licensing examination, and reviewing curriculum, continuing professional development, placement and rotation policy, and distribution of responsibilities.

## Background

Ethiopia has made an impressive progress in improving the health of its people in the last few decades. It has improved maternal and child survival, reduced burden of HIV/AIDS, malaria and tuberculosis. It has also extended longevity of its people. The country is on the right path to population age structure that enables demographic dividend [1, 2]. These gains have partly been made possible through huge investments in the health system. For instance, the number of public hospitals and health centers increased from 2782 in 2010 to 3736 in 2015 [3, 4]. The total health workforce density also increased from 0.64 to 1.63 per 1000 population between 2003 and 2015 [5]. The national health workforce density is however far lower than the 2.28 doctors, nurses and midwives per 1000 population threshold set by World Health Organization [6].

Despite improvements in the health systems, the population of Ethiopia still bears a heavy brunt of morbidity and mortality. The maternal mortality ratio (353/100,000) and under-five mortality rate (59.2/1000) are unacceptably high [7]. Ethiopia is one of the high burden countries for HIV/AIDS, tuberculosis and neglected tropical diseases [2]. Non-communicable diseases cause 23,118.1 disability-adjusted life years (DALYs) lost per 100,000 population [8].

The health sustainable development goals (SDGs) and Ethiopia’s health sector transformation plan include broad targets, placing greater demand on ability of the national health system to ensure universal health coverage. Needless to say adequate and well-performing physician workforce would advance efforts towards the universal health coverage.

On the numeric front, the last decade saw massive improvements in the production capacity. The number of medical schools and annual graduation outputs leapfrogged from 7 to 35 [5] (5-fold increase) and 162 to 2100 (13-fold increase) [4], respectively. The increment in doctors’ stock enabled the country to reach 1:17,720 physician to population ratio [5]. However, echoing with global scenario, there was unequal distribution of medical schools and doctors between the rural and urban settings and across the geographic regions. Communities in the rural and remote areas were particularly underserved which clearly needs urgent actions [9, 10].

Unless these newly deployed doctors mastered all essential competencies including for clinical procedures, health programing and research roles, the increment in the numbers alone would have limited impact on population health. Little was known about performance of the junior doctors other than their production and distribution thus far. To the best of our knowledge, there was only one published study in the past decade which showed junior doctors in Ethiopia had competency gaps to do clinical procedures and public health tasks [11]. Moreover, as part of its effort to ensure quality of healthcare, the Ministry of Health was working to establish national licensing examination for university graduates. Practice, task or job analysis can be used to assess needs for and gaps in education and training, update scope of practice, optimize workforce deployment, and develop blueprint for licensing examinations [12–14]. Hence, we conducted a practice analysis study to identify gaps in medical education and practice; and to determine composition of subjects in the licensing examination for medical undergraduates.

## Methods and materials

### Study design and sample

We conducted a cross-sectional study in February 2015 to assess practice of junior doctors (best known as general practitioners in Ethiopia) working at public hospitals. Only doctors with 6 months to 4 years of work experience were eligible to participate. The rationale for the lower limit of work experience was that practicing for at least 6 months would give opportunity for the junior doctors to have reasonable exposure in various service areas and to provide valid judgments about their medical practice. The cap was made four years because we assumed that with more than four years of experience, the ability of doctors to characterize their preservice education would be limited as some competencies might be learned on the job. The doctors might also forget competencies learned in the preservice education over longer time. We calculated the sample size to be 198 using a single population proportion formula and based on 95% level of confidence, maximum variability of attributes with proportion of 0.5, plus or minus 15% points of relative error, a design effect of 1.2, and a non-response rate of 10%. Since the number of junior doctors working in public hospitals in 2013/2014 [15, 16] was estimated to be 1431, we used a finite population adjustment. We used a two-stage stratified cluster sampling method to select study participants. The 9 regional states and 2 city administrations were the strata and the public hospitals were the clusters. We selected hospitals and doctors randomly from the lists of respective sampling units. Expecting to find at least 3 junior doctors in one hospital, we decided to select 66 out of the total 127 functional public hospitals. We allocated the 66 hospitals to the regions proportionally. A power allocation technique was used to find the optimum number of hospitals for the regions with few hospital numbers. Data collectors received a list of junior doctors at each hospital. If the number of junior doctors at the time of survey was three or less, the data collectors invited all junior doctors to participate. If the number of doctors was greater than three, they randomly selected three.

### Data collection

We drafted a preliminary list of tasks (competencies) for junior doctors using the national scope of practice, local curricula and international competency frameworks. The draft list was reviewed and validated in an expert panel workshop representing medical educators, practitioners and health programmers. The final 222 tasks were included in a structured questionnaire. The respondents made judgments on the tasks identified in the list. We collected data on three variables. The first measurement was frequency: how often a respondent performs a task with exclusive response options of daily, weekly, monthly, rarely and never. The second one was competence: how comfortable a respondent is to do a task with exclusive response options of proficient, competent and not capable. The third was the importance: how important a task is for patient or public health outcomes with exclusive response options of high, moderate and low. Open-ended questions were added to capture other tasks performed by the doctors, and to identify tasks that they were unable to perform due to lack of resources. Background data on the respondents were also collected. To determine composition of subjects in a blueprint for the national licensing examination, we collected additional data from 36 practicing doctors and experts so that it can be triangulated with findings of this study. After explaining the purpose and process, the panel were given time to look into the tasks and categories. We then asked them to individually allocate percentage weights to different subjects based on professional judgement and local context. The experts considered the importance and frequency of doing tasks for addressing the health needs of the population. We believed that incorporating experts’ judgements on relative weights of subjects in licensing examination would enhance validity of the examination. To assure data quality, data collectors were trained for three days on study instrument and data collection procedures. The data collection process was supervised and errors found were corrected timely.

### Data analysis

We cleaned, coded, and fed the collected data into EPidata version 2.0.2. After data entry, the data were re-cleaned to correct errors. We calculated proportions using SPSS version 22 to identify distribution of the tasks with regard to the three variables: importance, frequency, and competence. We summarized results using average statistics (mean and median), graphs and tables. To develop the exam blueprint, we used proportions and means on task frequency and importance from this study. We also considered judgement of experts on proportions of examination items from each subject. In the first step, we organized the tasks into six subjects which are found in undergraduate medical education; namely, internal medicine, pediatrics, surgery, obstetrics and gynecology, minor clinical specialties and public health. The minor clinical specialties included psychiatry, ophthalmology, dermatology, dentistry, and ear, nose and throat. Secondly, we summed up mean frequency (range: 1–5) and mean importance (range: 1–3) to generate a composite score for each competency (range: 2–8). Thirdly, we aggregated the composite scores of all competencies in a category to calculate average score for each category. Fourthly, we calculated proportions for each category out of the total (the sum of average scores of all categories). We further distributed the proportion of the minor clinical specialties category to each minor subject based on relative weights. We calculated the relative weights using average scores of the minor subjects (step 3), subject proportions out of minor subjects’ total (step 3) and average score of the minor specialty category (step 4). In the fifth step, we calculated the average of expert ratings for each subject. Finally, we computed average of percentages obtained in steps 4 and 5 to determine share of exam items for each category.

### Ethical consideration

We obtained an ethical clearance for this study from the Johns Hopkins School of Public Health Institutional Review Board. The Ethiopian Ministry of Health also approved the study protocol and wrote support letters to the hospitals. The study team members met with the hospital administrations to explain purpose of the study and obtained permission. The data collectors obtained an oral informed consent from each study participant.

## Results

### Description of study participants

A total of 191 junior doctors participated in the study. The response rate was 96.5%. Most respondents were males (74.6%), were 25–29 years of age (92.6%), and had less than 2 years of work experience (69.8%) (Table 1).

**Table 1:** Socio-demographic characteristics of study participants, Ethiopia, 2015

Socio-demography characteristics	Number of Participants (%)
Sex (*n* = 189)	
Male	141 (74.6)
Female	48 (25.4)
Age (*n* = 189)	
20–24	8 (4.2)
25–29	175 (92.6)
30–34	6 (3.2)
Type of hospital (*n* = 191)	
Non-teaching	150 (78.5)
Teaching	41 (21.5)
Level of hospital (*n* = 191)	
Referral	56 (29.3)
General	69 (36.1)
Primary	66 (34.6)
Setting (*n* = 186)	
Urban	165 (88.7)
Rural	21 (11.3)
Work experience (*n* = 189)	
Less than two years	132 (69.8)
2–4 years	57 (30.2)

### Perceived importance

**Fig. 1:**
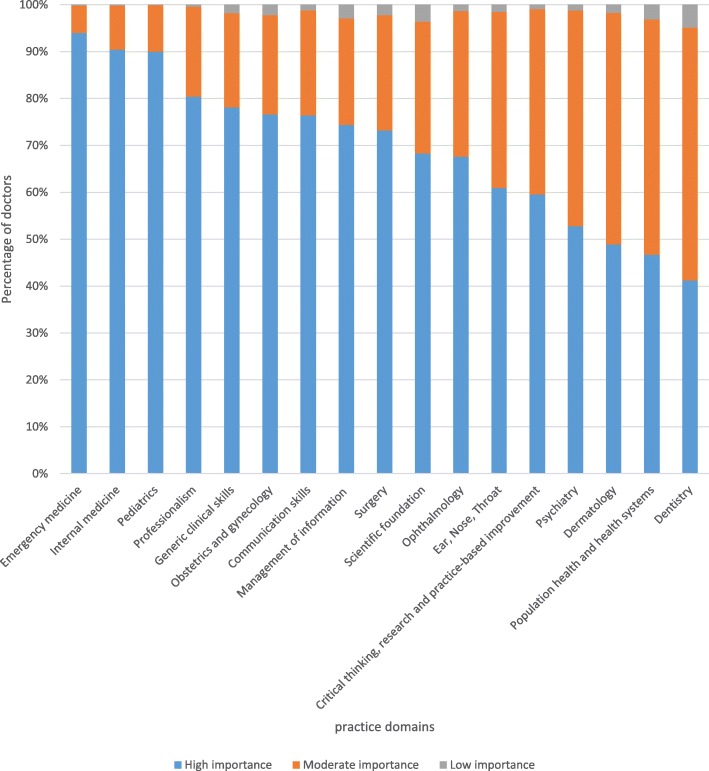
Junior doctors’ perceptions about importance of tasks summarized by practice domain, Ethiopia, 2015 (*N* = 191)

Figure 1 presents respondents’ perceived importance of tasks summarized by the practice domains. Nearly all respondents believed tasks included in the survey were moderately to highly important for a patient or population health outcome. The most popular practice domains were emergency medicine, internal medicine and pediatrics which were deemed highly important by about 170 (90%) respondents. In contrast, fewer than half of the respondents rated dentistry, public health and dermatology domains as highly important. In terms of specific tasks, performing vasectomy, tubal ligation and male circumcision were rated as low importance by 37(19.4%), 25 (13.1%) and 25 (13.1%) respondents, respectively.

### Practice patterns

We examined practice patterns by applying different analysis techniques. First, we identified clinical services performed frequently. Accordingly, the top five routine tasks done by more than 151 (80%) doctors were diagnosing and managing urinary tract infection, diagnosing and managing hypertension, managing pneumonia in children, managing diarrhea and vomiting in children, and managing childhood fever. (See Table 2).

**Table 2:** Top 30 patient management problems encountered by junior doctors, practice analysis study, Ethiopia, 2015 (*N* = 191)

Patient management problems	Perform every day (%)	Perform weekly but not daily (%)	Total (%)
Diagnose and manage urinary tract infection	75.9	19.4	95.3
Diagnose and manage hypertension	52.4	34.6	86.9
Manage pneumonia of children	64.4	19.9	84.3
Manage diarrhea and vomiting for children	62.8	20.9	83.8
Mange childhood fever	59.2	22.0	81.2
Diagnose and manage anemia	50.5	30.5	81.1
Diagnose and treat pulmonary and extra pulmonary tuberculosis	37.7	39.8	77.5
Diagnose and manage different forms of diabetes mellitus	35.6	40.8	76.4
Diagnose and manage bronchial asthma	35.6	39.8	75.4
Diagnose and manage shock of different forms	29.8	45.0	74.9
Manage trauma	42.4	32.5	74.9
Diagnose and manage acute exacerbation of asthma	25.1	45.0	70.2
Diagnose and manage congestive heart failure	29.3	40.8	70.2
Offer an HIV test and counsel for HIV	51.8	17.8	69.6
Diagnose and manage hypertensive urgency and emergency	28.3	40.3	68.6
Diagnose and manage children with protein energy malnutrition	41.9	25.1	67.0
Diagnose acute abdomen	28.8	37.2	66.0
Diagnose and treat malaria	35.1	29.3	64.4
Diagnose and manage fracture	24.1	39.3	63.4
Diagnose and manage diabetic ketoacidosis	20.9	41.9	62.8
Diagnose and treat STIs (genital ulcer, discharge and mass)	24.6	37.7	62.3
Diagnose and manage common skin infections like cellulitis and impetigo	31.4	28.8	60.2
Diagnose and manage otitis media	17.3	41.4	58.6
Manage head injury	17.3	40.8	58.1
Diagnose and manage common allergic skin disorders	22.5	34.0	56.5
Diagnose and manage common fungal skin infections	23.6	30.9	54.5
Diagnose and manage coma of different causes	19.9	32.5	52.4
Diagnose and manage tonsillitis and peritonsillar abscess	21.5	28.8	50.3
Diagnose and manage chronic liver disease	20.0	25.8	45.8
Diagnose and manage conjunctivitis	14.1	31.4	45.5

Secondly, engagement in practice domains was analyzed by calculating proportion of doctors who performed one or more tasks in a domain on weekly basis. Consequently, more than 151 (80%) doctors were engaged in the following domains: internal medicine, public health, emergency medicine, surgery and pediatrics. In contrast, less than 113 (60%) doctors were engaged in dentistry, ophthalmology and psychiatry. (See Table 3).

**Table 3:** Percentage of junior doctors performing one/more tasks in a domain on weekly basis, Ethiopia, 2015 (*N* = 191)

Practice domain	Percent
Internal medicine	98.4
Public health	97.9
Emergency medicine	96.9
Surgery	91.1
Pediatrics	89.5
Obstetrics & gynecology	75.3
Dermatology	71.7
ENT	66
Dentistry	52.9
Ophthalmology	52.4
Psychiatry	17.3

**Fig. 2:**
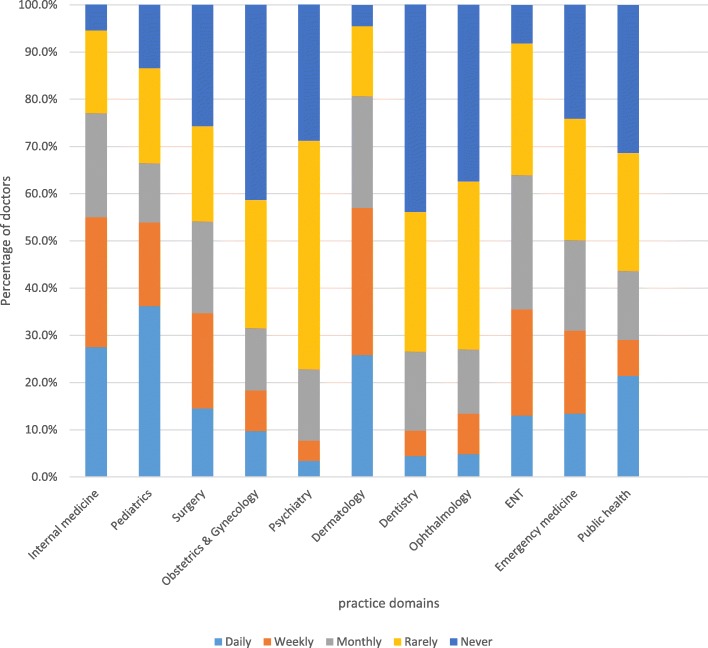
Median performance frequency of tasks summarized by practice domain, Ethiopia, 2015 (*N* = 191)

Thirdly, since the second approach did not consider all tasks in a domain which might overestimate engagement, we calculated median. Accordingly, dermatology, internal medicine, and pediatrics were the three most common practice domains, in which more than 94 (50%) doctors performed the tasks on daily or weekly basis. However, psychiatry, ophthalmology, dentistry, and obstetrics & gynecology tasks were not done even once in a month time by majority of the respondents. (See Fig. 2).

We also identified clinical procedures and public health tasks that were never conducted by many respondents. More than 70% had never inserted or removed a long-acting reversible contraceptive device (IUD or implants), 83.8% of respondents had never done an appendectomy, and 95.5% had never done a vasectomy. Similarly, more than 40% of respondents never participated in research. (See Table 4).

**Table 4:** Top 30 competence gaps and percentage of respondents who never performed those tasks, Ethiopia, 2015 (*N* = 191)

Competencies	% not capable	% never done the task
Perform vasectomy	82.7	95.5
Perform tubal ligation	77.0	90.6
Perform VIA (Visual Inspection with Acetic Acid)	74.1	89.5
Perform appendectomy	73.3	83.8
Perform cesarean section	70.7	81.2
Perform dental extraction	69.1	88.0
Diagnose retinal detachment	58.1	82.7
Perform culdocentesis	58.1	81.7
Perform excisional biopsy	45.0	70.7
Perform and interpret AFB (Acid Fast Bacilli)	41.9	71.2
Perform hydrocelectomy	41.4	67.0
Insert and remove IUD	39.3	79.1
Perform and interpret peripheral morphology	35.6	57.6
Insert and remove implants	34.6	72.8
Diagnose glaucoma	33.5	61.8
Perform and interpret stool microscopy	32.5	64.9
Perform and interpret blood film	30.9	62.3
Prepare proposals for funding	27.4	70.0
Perform MVA (manual vacuum aspiration) for endometrial biopsy	23.6	64.9
Perform circumcision	23.6	49.7
Perform arthrocentesis	23.0	44.5
Evaluate policies, programs, and services	22.1	42.6
Use evidence in developing, implementing, evaluating, and improving policies, programs, and services	20.6	44.2
Develop program and/or project goals and objectives	20.5	45.3
Apply low lying forceps	19.9	60.7
Perform vacuum assisted delivery	19.4	56.5
Analyze and interpret quantitative and qualitative data	19.1	53.4
Manage ophthalmic chemical burns	18.3	62.3
Justify programs for inclusion in organizational budgets	18.1	50.0
Justify programs for inclusion in organizational budgets	18.1	50.0

One hundred seventy two (91.1%) respondents reported shortage of supplies affected their performance. The most frequently mentioned supply gaps were related to medical equipment (85.1%), diagnostic facilities (81.6%) and pharmaceuticals (21.8%).

### Top competence gaps

We identified the top 30 competence gaps which primarily included procedural skills in surgery, obstetrics & gynecology, family planning and clinical laboratory. More than 132 (70%) respondents said they were unable to perform appendectomy, cesarean section, vasectomy, tubal ligation, and cervical cancer screening. Respondents also acknowledged gaps in the areas of program, policy and research. (See Table 4).

We also analyzed responses to open-ended questions about training gaps. About 103 (54.5%) reported that their education did not adequately prepare them for some tasks expected from them. The most frequently mentioned gaps pertained to doing emergency surgeries (mainly cesarean section and appendectomy), research, clinical procedures, laboratory procedures, and public health activities, in descending order.

### Blueprint for a national licensing examination

Analysis of junior doctors’ perceptions of importance and frequency of tasks suggested composition of licensing examination to be 21.6% internal medicine, 17.6% pediatrics, 16.8% surgery, 15.2% obstetrics and gynecology, 14.5% public health, 3.4% dermatology, 3% ear, nose and throat, 2.9% dentistry, 2.6% psychiatry and 2.4% ophthalmology. However, experts group allocated notably higher percentage for psychiatry, dermatology, ophthalmology, ear, nose and throat, and dentistry, but lower percentage for public health. Taking into account results from both groups, the highest percentage weights went to internal medicine, pediatrics, surgery, obstetrics and gynecology, and public health, which collectively accounted for 81.8% of exam questions. Ophthalmology, psychiatry, dentistry, ear, nose and throat, and dermatology comprised the remaining 18.2% of the questions. (See Table 5).

**Table 5:** Recommendations for national medical licensing examination composition, practice analysis study, Ethiopia, 2015

Practice Area	% (Junior doctors)	% (Experts)	Composite
Internal Medicine	21.6%	20.2%	20.9%
Surgery	16.8%	14.8%	15.8%
Pediatrics	17.6%	16.9%	17.2%
Gynecology & Obstetrics	15.2%	15.8%	15.5%
Public Health	14.5%	9.5%	12%
Psychiatry	2.6%	5.3%	3.9%
Dermatology	3.4%	4.8%	4.1%
Ophthalmology	2.4%	4.6%	3.5%
Ear, Nose and Throat	3%	4.4%	3.7%
Dentistry and Oral health	2.9%	4%	3.5%

Note: Total may be slightly over 100% due to rounding

## Discussion

### Variable participation of junior doctors in all practice domains

Our study found out that practice of junior doctors was reasonably comprehensive providing services to wide range of patient problems and participating in public health roles. There was, however, considerable variation among practice domains. Internal medicine and paediatrics tasks were the most frequently performed activities. In contrast, psychiatry, dentistry, ophthalmology, and obstetrics and gynaecology tasks were not routinely done by the junior doctors. This may be a reflection of disease epidemiology in Ethiopia where the top causes of hospital visits and admissions are medical and paediatrics problems [4]. Our results may also be due to limited opportunities for practice due to presence of other health workers primarily tasked with such services. For example, if available, midwives and obstetricians are the main providers of obstetrics services. Similarly, mid-level health workers (instead of junior doctors) are often assigned to psychiatry, dental and eye clinics. Short service duration of junior doctors before residency training may also limit their chance to rotate and practice in various departments [16]. These findings invite important questions. How are placement decisions made? Should hospitals review placements and rotations to ensure full utilization of junior doctors as general practitioners? Should we be concerned about erosion of competencies for rarely conducted services? Should there be drills or rotations to maintain competence in rarely performed tasks? As junior doctors are increasingly confined to services related to internal medicine and paediatrics unlike previous times, they might encounter skill fade for rarely used competencies. This might strain primary care especially if they were reassigned to provide care in rural areas where there were no specialists. Similar concerns have been reported from other countries. A study in United Kingdom reported that foundation doctors were routinely conducting internal medicine and pediatrics tasks but, rarely did surgical and laboratory procedures [17]. Another study in Western Europe reported that general practitioners had gate keeping roles and wider scope of work [18]. A Portugal based study found out that over 80% of new doctors had managed few surgical, obstetric, gynaecologic and emergency patients [19]. A study from Vietnam showed that psychiatry, ophthalmology, and gynecology and obstetrics were infrequently performed [20]. Other researchers also reported that non-specialist doctors did not routinely provide care to patients with mental [21] and dental problems [22].

### Skills gap for clinical procedures and public health tasks

Many junior doctors had never carried out clinical procedures, health programming tasks and researches. In Ethiopian context, the findings might not be surprising as these tasks were primarily conducted by other health workers. Infrequent performance could also be due to the presence of significant gaps in the training curricula of the junior doctors. There was an empirical evidence showing competence gaps and its concordance with infrequent performance [24]. However, we cannot help asking why junior doctors’ scope of practice and curricula included tasks primarily performed by other health workers. One should identify the pros and cons of task sharing. The scope of general practice needs to be clear in a way that junior doctors should do everything or focus on things other health workers do not conduct. It also requires to assess ability of junior doctors to provide comprehensive services. This suggests a careful examination to assess the alignment of the policy (scope of practice and curricula) with real practices of junior doctors. Redefining competencies of junior doctors based on real practice by medical educators and reviewing their scope of practice by regulators merit consideration.

These findings also warrant increasing emphasis on skills training to medical students through use of clinical simulations and adequate clinical practice opportunity to ensure skills mastery. Medical educators should also ensure that medical students master essential competencies for designing and implementing researches and health programs. These can be achieved through increased community-based education and work-based learning. There is also a need for strengthening in-service training and clinical mentorship programs for newly deployed doctors. Similar to the results of our study, a prior study in Ethiopia showed that many junior doctors had competency gaps to conduct clinical procedures and public health roles [11]. A study in Vietnam showed that many doctors never performed procedural skills and public health tasks [20]. A study in Australia also found out that 80% of doctors had not conducted any surgical, and obstetric and gynecologic procedures recently [23]. A study in Nepal also recommended continuing professional development for doctors on procedural skills and public health tasks [25]. However, our findings differ from a Portuguese study which claimed that doctors demonstrated sufficient level of procedural and public health competencies [19].

### Unfavorable perceptions of task importance for some practice domains

The tasks of emergency medicine, internal medicine, pediatrics, surgery, and obstetrics and gynecology were highly important for many respondents. Many of the junior doctors, however, did not consider the tasks in public health, dentistry, ophthalmology, psychiatry, dermatology, and ear, nose and throat as highly important. In addition, many respondents did not consider family planning and male circumcision as highly important which might explain why they never performed these tasks. These findings are comparable with results from Vietnam and Nepal [20, 25]. However, studies from Nigeria and USA had reported that doctors recognized importance of public health tasks [26, 27]. Other researchers had also reported that appreciation of managing mental, dental and skin problems [28–30]. Considering that doctors may not make an effort to learn and perform tasks they do not deem important, research is needed to understand why junior doctors did not consider some practice domains critical and its implications.

### Improving validity of licensing examinations

Last but not least, results of this study in deciding composition of national licensing examination were largely consistent with the suggestions of expert judgments. However, presence of some differences required combination of both methodologies which probably provides more valid reflection of relative weights of subjects in the examination. Our recommendation is supported by similar approaches in designing licensing examination for graduates of health science programs [12–14]. We recommend conducting practice analysis periodically to decide distribution of national licensing examinations.

## Limitation

One of the strengths of this study is that it had a national coverage of junior doctors working at various levels of public hospitals. Including junior doctors working in private health facilities, non-governmental organizations, and program management positions might have given additional perspectives. However, we know this accounts for a small proportion of the junior doctor population. Although practice analysis is a powerful and efficient methodology to identify performance gaps, descriptive nature of the study did not allow it to identify causes of performance gaps.

We acknowledge that self-assessment of competency is not the most reliable measure. Errors and subjectivity can affect its accuracy if precautions are not taken. However, effective self-assessment is the mainstay of medical profession as it can strengthen medical education, practice and regulation [31]. In this study, we improved quality of self-assessment through training of data collectors, informing respondents about the study purpose, providing adequate time for self-reflection and ensuring anonymity.

We also recognize that cross-sectional study based on self-report is susceptible to recall and social desirability biases. While it was possible that respondents did not completely and accurately remember performance frequency, we do not believe the deviations were non-random and significant enough to impact the overall practice pattern. Similarly, while respondents may have overestimated their capability knowingly (due to social desirability) or unknowingly (not understanding the required level of performance), our study uncovered substantial competence gaps, making it a less important concern.

## Conclusions

Practice of junior doctors was comprehensive in managing patient problems and assuming public health roles. However, there was considerable variation among practice domains. The major concern is that junior doctors rarely managed psychiatry, ophthalmology and dental patients. The doctors also had competence gaps in procedural, research and health programming skills. These substantial performance gaps demand reviewing medical education curricula and continuing professional development opportunities. Further examining the infrequent performance and unfavorable importance of tasks is needed to refine job placement and rotation policy, and to decide on redistribution of responsibilities. The results of practice analysis are used to improve standards of national licensing examinations.

## Abbreviations

AFB: Acid Fast Bacilli
DALYs: Disability adjusted life years
ENT: Ear nose throat
FMOH: Federal Ministry of Health
HIV/AIDS: Human immunodeficiency virus/ Acquired immunodeficiency syndrome
IUD: Intra uterine device
MVA: Manual vacuum aspiration
SDGs: Sustainable development goals
SPSS: Statistical Package for the Social Sciences
STIs: Sexual transmitted infections
UK: United Kingdom
